# Reliability of Tethered Swimming Evaluation in Age Group Swimmers

**DOI:** 10.2478/hukin-2014-0043

**Published:** 2014-07-08

**Authors:** Nuno Amaro, Daniel A Marinho, Nuno Batalha, Mário C Marques, Pedro Morouço

**Affiliations:** 1 Research Centre for Human Movement Sciences, Polytechnic Institute of Leiria, Leiria, Portugal.; 2 Research Centre in Sports Sciences, Health and Human Development, Covilhã, Portugal.; 3 University of Beira Interior, Covilhã, Portugal.; 4 University of Évora, Évora, Portugal.; 5 Centre for Rapid and Sustainable Product Development, Polytechnic Institute of Leiria, Leiria, Portugal.

**Keywords:** swimming, training and testing, propulsive force, front crawl

## Abstract

The aim of the present study was to examine the reliability of tethered swimming in the evaluation of age group swimmers. The sample was composed of 8 male national level swimmers with at least 4 years of experience in competitive swimming. Each swimmer performed two 30 second maximal intensity tethered swimming tests, on separate days. Individual force-time curves were registered to assess maximum force, mean force and the mean impulse of force. Both consistency and reliability were very strong, with Cronbach’s Alpha values ranging from 0.970 to 0.995. All the applied metrics presented a very high agreement between tests, with the mean impulse of force presenting the highest. These results indicate that tethered swimming can be used to evaluate age group swimmers. Furthermore, better comprehension of the swimmers ability to effectively exert force in the water can be obtained using the impulse of force.

## Introduction

There are several factors that affect swimmers’ performance such as: swimming technique, strength and physiological measures. Among these, force exerted in water is a major factor that influences success in swimming (Keskinen et al., 1989; Girold et al., 2007; Barbosa et al., 2010) and its importance is higher as the swimming distance diminishes (Stager and Coyle, 2005; Morouço et al., 2011a). Thus, the measurement of swimming propulsion is of great interest to sports biomechanics, therefore its evaluation is highly complex (Payton et al., 2002; Marinho et al., 2011). In order to determine the force exerted by a swimmer in an identical context to the competition (i.e. in water), tethered swimming has been one of the most frequently used methodologies in the field of biomechanics (Akis and Orcan, 2004).

In the study by Magel (1970), a polygraph was used to characterize the four swimming techniques of 26 highly trained college swimmers along a tethered swimming test of 3 minutes. The author found that high levels of force production could be achieved in shorter durations of tethered swimming and that the measurement of these forces could be a reliable indicator to estimate the force produced during free swimming. Furthermore, Yeater et al. (1981) conducted an experiment using fully tethered swimming with 18 male athletes. Positive correlations were found between mean tethered force and velocity in front crawl and negative correlations between crawl velocity and the peak/mean force ratio. Since the study of Yeater et al. (1981), several investigations have shown significant relationships between tethered forces and swimming velocity (e.g. Keskinen et al., 1989; Dopsaj et al., 2000; 2003), differing according to age and maturity (Vorontsov et al., 1999; Taylor et al., 2001), competitive level (Sidney et al., 1996) and swimming distance (Yeater et al., 1981; Morouço et al., 2011a).

Nowadays, technological improvements allow an easy and operative way of assessing individual force - time curves (Toubekis et al., 2010), which seems to be a reason for considering tethered swimming as a useful and reliable methodology for the evaluation and control of swimmers training (Dopsaj et al., 2003; Kjendlie and Thorsvald, 2006). It evaluates aerobic (Pessôa-Filho and Denadai, 2008) as well as anaerobic (Ogonowska et al., 2009; Morouço et al., 2012) energetic profiles, with similar muscular activity (Bollens et al., 1988) and oxygen consumption (Lavoie and Montpetit, 1986) as in free swimming. Although it may induce some kinematic changes (Maglischo et al., 1984; Psycharakis et al., 2011), it is assumed that the force produced in this test is similar to the force required to overcome the drag in freestyle swimming (Dopsaj et al., 2000; 2003; Morouço et al., 2014). However, swimming with no displacement and the effort induced by this test could affect the results. Hence, it is recommended that swimmers have some experience in tethered swimming and they should be given the opportunity to be familiarized with the test procedures before an evaluation (Psycharakis et al., 2011). Evidence about the familiarization with the test procedures in previous studies is scarce. Thus, those results could have been underestimated by the initial difficulty of familiarization with the test.

Several studies have used different measures of force production in tethered swimming tests such as: average force (Ria et al., 1990; Taylor et al., 2001; Morouço et al., 2011a), average of maximum force (Yeater et al., 1981; Fomitchenko, 1999), peak maximum force (Christensen and Smith, 1987; Keskinen et al., 1989), impulse of force (Dopsaj et al., 2000; Dopsaj et al., 2003; Morouço et al., 2014) and fatigue index (Morouço et al., 2012) which has spawned controversy about which one could be more associated with performance. Taylor et al. (2001) concluded that only average force was a reliable parameter to associate with swimming velocity in age group swimmers. On the opposite, Dopsaj et al. (2000) and Morouço et al. (2014) concluded that the impulse of force had a better relationship with swimming performance. These discrepancies led us to question whether the measures to be assessed could differ depending on the swimmers’ level or if they were a result of the lack of evaluation of the impulse of force (Taylor’s et al., 2001). If one considers that propulsion may occur along the whole underwater phase of the stroke (Marinho et al., 2011) and not only in one specific moment (maximum force) and if a lower amount of force applied during a longer period can mean equal or further advancement of the swimmer, then the impulse of force should be considered. These inconsistencies reveal the need for further studies to clarify the methodological options. Additionally, it is clear in the literature that most studies with tethered swimming tested high level or elite swimmers. Thus, it is crucial to understand whether this methodology is reliable and provides benefits to age group swimmers whose technique development is still scarce.

Therefore, the aim of the present study was to examine the reliability of tethered swimming evaluation with age group swimmers. It was hypothesized that, as in adult swimmers, tethered swimming can be used as a reliable methodology to evaluate age group swimmers.

## Material and Methods

### Participants

The study involved 8 male swimmers that volunteered for the experiment (age 15.3 ± 1.17 years; body height 1.68 ± 0.06 m; body mass 57.2 ± 9.93 kg; span 1.70 ± 0.06 m. The personal best for the 50 m freestyle long course was 28.59 ± 1.47 s. The subjects had at least 4 years of experience in competitive swimming participating in national level competitions. No swimmer suffered from any illness or any other restrictions that could hinder their performance during the tests. All procedures were in accordance with the Declaration of Helsinki in respect to human research. All subjects and their parents gave their consent and the study was approved by the Scientific Committee of the University of Beira Interior.

### Apparatus

**Figure 1:**
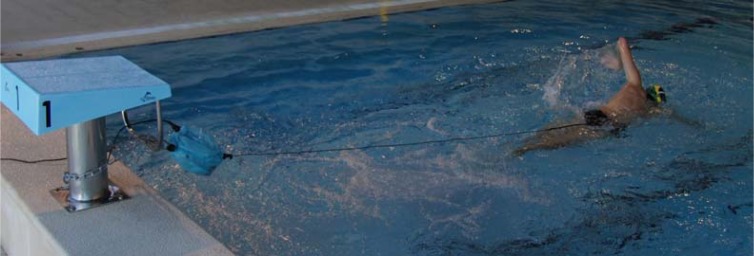
Experimental apparatus of the tethered swimming tests

The testing apparatus consisted of a load-cell system (Globus™, Codognè, Italy) recording at 100 Hz with a measurement capacity of 4903 N. The load-cell was connected by a cable to a Globus Ergometer data acquisition system (Globus™, Codognè, Italy) that exported the data in ASCII format to a PC. The load-cell was attached to the starting block (Figure 1) through a chain locked with a certified aluminum carabiner (Petzl CE EN 362, CE EN 12275, type K - major axis strength: 28 kN). It was proofed and tested prior to testing and between tests. The load-cell calibration was verified with the use of 5 kg, 10 kg and 20 kg standard weights. Subjects were wearing a nylon belt attached to a steel cable with a certified aluminum carabiner (Petzl CE EN 362, CE EN 12275, type K - major axis strength: 28 kN) with 3.5 m length (0.5 cm diameter). The attachment of the load-cell to the starting block created a 5.7° angle in relation to the water surface.

### Procedures

Before tests and aiming to familiarize subjects with the methodology, several training sessions had been conducted during which the subjects engaged in different tethered swimming exercises with various intensities and durations.

For test 1, after a 1000 m moderate intensity warm-up (400 m swim, 100 m pull, 100 m kick, 4 × 50 m at increasing speed, 200 m easy swim) each subject executed a maximal intensity front crawl tethered swimming test. Preceding the starting signal, swimmers adopted a horizontal position with the cable fully extended starting the data collection only after the first stroke cycle was completed. This procedure was used to avoid the inertial effect of the cable extension usually produced immediately before or during the first arm action (Morouço et al., 2011a). The duration of the exercise was 40 s with an initial phase of 10 s with moderate intensity and 30 s at maximum intensity. Participants were told to follow the breathing pattern they would normally apply during a 50 m front crawl event, and were verbally encouraged throughout the tests to maintain maximal effort over the duration of the tests. The end of the test was marked through an acoustic signal. Twenty four hours later, for test 2, the same experimental procedures were conducted with the same conditions.

Experiments were carried out during a competitive period to ensure that the subjects were in a prime training period. All tests occurred in the same 25 m indoor swimming pool (27 – 28° C of water temperature).

### Data Analysis

Tethered swimming data were exported to a signal processing software (AcqKnowledge v.3.7. Biopac Systems, Santa Barbara. USA) to assess the individual curves of force (y axis) along time (x axis). Data were filtered with a 4.5 Hz cutoff low-pass according to residual analysis (residual error versus cut-off frequency). As the force vector in the tethered system presented a small angle in relation to the water surface, data were corrected computing the horizontal component of force (Taylor et al., 2001). The following measures were estimated for each participant: maximum force (maxF) as the higher value obtained in individual force-time curve; mean force (meanF) as the mean of F values registered along the 30 s; mean impulse of force (impF) as the quotient of the sum of single-stroke impulse and the number of strokes performed in the 30 s.

### Statistical analysis

Descriptive statistical analysis was used for the calculation of test/retest mean values (mean), standard deviation (SD), minimum measure value (min), maximum measure value (max) and coefficient of variation (cV%) for all measures. The normality assumption was checked by Shapiro Wilk tests (SW), thus parametrical statistics analyses were applied. Relative and absolute reliability were calculated through the Intraclass Correlation Coefficient (ICC) and Coefficient of Variation (cV%), respectively. General reliability was calculated using Cronbach’s alpha for internal consistency of measures and the Bartlett’s Test of Sphericity as a measure that determines the homogeneity of variances. SPSS for Windows® (version 20.0, Chicago, IL, USA) was used for all statistical procedures. The level of statistical significance was set at p < 0.05.

## Results

Table 1 contains the basic descriptive statistics results of both tests. Results of the 3 assessed measures were similar between the test and retest. The coefficient of variation which can be considered as a measure of descriptive homogeneity of raw results, ranged between 14.7% and 23.1%, and 17.6% and 24.4% for the test and retest, respectively.

Distribution of used measures did not differ from the model of hypothetically normal p values from 0.21 (Fmax) to 0.78 (ImpF) for the test, and 0.26 (meanF) to 0.71 (impF) for the retest.

Table 2 presents the results of single reliability of used measures. Cronbach’s alpha for the reliability among the used measures ranged from 0.970 for maximum force to 0.995 for the impulse of force. Results of the Bartlett’s Test of Sphericity showed that *x*^2^ was statistically significant in all measures (p<.0001). Intraclass Correlation Coefficient was *excellent* for all measures ranging from 0.942 for maximum force to 0.990 for the impulse of force.

## Discussion

The aim of the present study was to examine the reliability of tethered swimming evaluation with age group swimmers. Overall results showed that tethered swimming was a highly reliable methodology to evaluate age group swimmers in the water.

In regard to internal consistency of measures, results showed a very high agreement for all metrics. These data may be considered excellent, which is in accordance with previous studies conducted with older and more skilled swimmers (Kjendlie and Thorsvald, 2006; Dopsaj et al., 2003). For instance, Dopsaj et al. (2003) evaluated 10 high-level swimmers and obtained similar reliability values. Small biases may be due to the swimmers level, but also to the duration of the tests. With an increased duration (60 s), these authors emphasized the importance of swimming technique devaluing the importance of force. On the other hand, with a smaller duration, our swimmers were able to keep the effort closer to maximal intensity throughout all test duration. Aiming to investigate the test-retest reliability in a 10 s maximal tethered swimming test, Kjendlie and Thorsvald (2006) assessed the maximum force of 32 swimmers. These authors stated that subject variations were very small, obtaining Cronbach’s alpha of 0.992. This value is in accordance with the obtained data in the present study that also assessed the reliability of other measures (mean force and impulse of force). Thus, tethered swimming, which has been used with high-level swimmers, seems to be also a highly reliable procedure to evaluate age group swimmers.

Technique development and strength improvement have been two issues of major concern for swimming biomechanics over the years. For instance, Newton et al. (2002) reported that an optimum level of strength and swimming power is necessary for good performance. Vorontsov (2010) proposed that during the pubescent period (12–14 years for girls and 14–16 years for boys) maturation and all its implications provide an optimal biological background for development of the anaerobic energy system, maximal power, specific muscular endurance, and speed-strength abilities. However, in the development of youth swimmers, especially at younger ages (12 and 14 years old) training focuses specifically on improving swimming technique (Barbosa et al., 2013), relegating the physical condition to later stages. We could state that from this age on swimmers begin the stage of specialization in a swimming technique and/or in a swimming distance (Morouço et al., 2011b). As a result it is relevant to emphasize other measures, which include strength, seeking balance between the development of technique and the ability to effectively exert force in the water. Thus, tethered swimming may emerge as a support tool for coaches and researchers in this crucial stage of the swimmers’ career.

It is well known that force exerted in water is a major factor to enhance swimming performance (Barbosa et al., 2010). Therefore, several methodologies have been used to evaluate the force exertion that a swimmer can produce in the water. One of those methodologies uses a load-cell to register the forces that a swimmer exerts when tethered. However, the question about which measures should be considered in tethered swimming evaluations remains open. On the one hand, Taylor et al. (2001) concluded that only average force was a reliable parameter to associate with swimming velocity in age group swimmers, to the detriment of maximum force peaks. On the other hand, Dopsaj et al. (2000) stated that the average impulse of force had a better relationship with swimming performance in elite sprinters. In our experiment, consistency of the impulse of force was higher than consistency of maximum or mean force. As aforementioned, propulsion may occur along the whole underwater phase of the stroke (Marinho et al., 2011). In a recent study, Morouço et al. (2014) have showed that the impulse of force presents a linear relationship with free-swimming velocity. These authors indicated previous studies that only assessed the maximum force that a swimmer exerts in the water, underestimated the role of stroke force mechanics in swimming performance. Indeed, maximum force comprises information about a single point per stroke: when maximum force is reached. However, according to the integral of force with respect to time, propulsion can occur throughout the underwater phase of the stroke (Marinho et al., 2011) and lower force applied in a longer stroke can produce similar (or even higher) momentum change than a higher force applied in a shorter stroke. Our results indicate that, also for age group swimmers, the impulse of force is a feasible measure and should be taken in consideration.

This study has some limitations. First, a sample size of 8 swimmers does not assure an extensive generalizability. Second, swimmers had to be attached to the starting block by a steel cable, which produced a small angle in relation to the water surface. This clearly could lead to a change in the swimmer streamline. And third, the swimmers might have inhibited their leg kicking in an attempt not to touch the cable with their feet.

In conclusion, according to our results, the 30 s maximal intensity tethered swimming provides a reliable tool to evaluate age group swimmers. Thus, the current study provides promising results for the application of tethered swimming to the evaluation of age group swimmers, as well as remarks for future research in this area. Systematic evaluations throughout the season may be an operational procedure for coaches to examine the ability of their swimmers to exert force in the water. Finally, it is suggested to assess the impulse of force as a more reliable metric to analyze the tethered forces.

## Figures and Tables

**Table 1:** Basic descriptive statistics

**Measures**	Test	Mean	SD	Min	Max	cV%	SW Z ratio	SW p value
maximum force	1	220.66	50.94	165.69	300.99	23.08	0.886	0.214
	2	217.86	53.07	162.81	306.29	24.35	0.913	0.372
mean force	1	86.10	12.62	71.47	105.95	14.66	0.908	0.338
	2	86.92	16.15	68.93	111.62	18.58	0.895	0.261
impulse of force	1	77.68	12.77	61.11	96.43	16.44	0.957	0.783
	2	75.71	13.31	58.64	95.86	17.58	0.950	0.708

SD=standard deviation; Min=minimum; Max=maximum; cV%= Coefficient of variation; SW=Shapiro Wilk

**Table 2:** Results of single reliability of used measures

Measures	Cronbach’s alpha	BTS	ICC
maximum force	0.970	x^2^ = 12.038; p = 0.001	0.942
mean force	0.977	x^2^ = 19.135; p = 0.000	0.955
impulse of force	0.995	x^2^ = 22.060; p = 0.000	0.990

*Legend: BTS* = *Bartlett’s Test of Sphericity; ICC* = *Intraclass Correlation Coefficient*
